# Dopamine receptors and key elements of the neurotrophins (BDNF, CDNF) expression patterns during critical periods of ontogenesis in the brain structures of mice with autism-like behavior (BTBR) or its absence (С57BL/6 J)

**DOI:** 10.18699/vjgb-24-46

**Published:** 2024-07

**Authors:** P.D. Pravikova, M.A. Arssan, E.A. Zalivina, E.M. Kondaurova, E.A. Kulikova, I.I. Belokopytova, V.S. Naumenko

**Affiliations:** Institute of Cytology and Genetics of the Siberian Branch of the Russian Academy of Sciences, Novosibirsk, Russia; Institute of Cytology and Genetics of the Siberian Branch of the Russian Academy of Sciences, Novosibirsk, Russia; Institute of Cytology and Genetics of the Siberian Branch of the Russian Academy of Sciences, Novosibirsk, Russia; Institute of Cytology and Genetics of the Siberian Branch of the Russian Academy of Sciences, Novosibirsk, Russia; Institute of Cytology and Genetics of the Siberian Branch of the Russian Academy of Sciences, Novosibirsk, Russia; Institute of Cytology and Genetics of the Siberian Branch of the Russian Academy of Sciences, Novosibirsk, Russia; Institute of Cytology and Genetics of the Siberian Branch of the Russian Academy of Sciences, Novosibirsk, Russia

**Keywords:** autism, BTBR and С57BL/6 J mice, BDNF, CDNF, dopamine receptors, ontogenesis, hippocampus, frontal cortex, аутизм., мыши BTBR и С57BL/6 J, BDNF, CDNF, рецепторы дофамина, онтогенез, гиппокамп, фронтальная кора

## Abstract

Analysis of the mechanisms underlying autism spectrum disorder (ASD) is an urgent task due to the ever-increasing prevalence of this condition. The study of critical periods of neuroontogenesis is of interest, since the manifestation of ASD is often associated with prenatal disorders of the brain development. One of the currently promising hypotheses postulates a connection between the pathogenesis of ASD and the dysfunction of neurotransmitters and neurotrophins. In this study, we investigated the expression of key dopamine receptors (Drd1, Drd2), brain-derived neurotrophic factor (Bdnf), its receptors (Ntrkb2, Ngfr) and the transcription factor Creb1 that mediates BDNF action, as well as cerebral dopamine neurotrophic factor (Cdnf) during the critical periods of embryogenesis (e14 and e18) and postnatal development (p14, p28, p60) in the hippocampus and frontal cortex of BTBR mice with autism-like behavior compared to the neurotypical C57BL/6 J strain. In BTBR embryos, on the 14th day of prenatal development, an increase in the expression of the Ngfr gene encoding the p75NTR receptor, which may lead to the activation of apoptosis, was found in the hippocampus and frontal cortex. A decrease in the expression of Cdnf, Bdnf and its receptor Ntrkb2, as well as dopamine receptors (Drd1, Drd2) was detected in BTBR mice in the postnatal period of ontogenesis mainly in the frontal cortex, while in the hippocampus of mature mice (p60), only a decrease in the Drd2 mRNA level was revealed. The obtained results suggest that the decrease in the expression levels of CDNF, BDNF-TrkB and dopamine receptors in the frontal cortex in the postnatal period can lead to significant changes in both the morphology of neurons and dopamine neurotransmission in cortical brain structures. At the same time, the increase in p75NTR receptor gene expression observed on the 14th day of embryogenesis, crucial for hippocampus and frontal cortex development, may have direct relevance to the manifestation of early autism

## Abstract (ru)

Анализ механизмов расстройства аутистического спектра (РАС) является актуальной задачей в связи с широкой и постоянно растущей распространенностью этого состояния. Исследование критических периодов нейроонтогенеза представляет интерес, поскольку манифестацию РАС нередко связывают с внутриутробными нарушениями развития головного мозга. Одна из перспективных на сегодняшний день гипотез постулирует связь патогенеза РАС с дисфункцией нейротрансмиттерных и нейротрофических систем. В настоящей работе исследована экспрессия генов ключевых рецепторов дофамина (Drd1, Drd2), нейротрофического фактора мозга (Bdnf), его рецепторов (Ntrkb2, Ngfr) и опосредующего действие BDNF транскрипционного фактора Creb1, а также дофаминового нейротрофического фактора (Cdnf) в периоды эмбриогенеза (e14 и е18) и постнатального развития (р14, р28, р60) в гиппокампе и фронтальной коре мышей BTBR с аутистизм-подобным поведением по сравнению с нейротипичной линией С57BL/6 J. У эмбрионов BTBR на 14-й день пренатального развития в гиппокампе и во фронтальной коре установлено увеличение экспрессии гена Ngfr, кодирующего рецептор p75NTR, трансдукция сигнала которого в эмбриогенезе приводит к активации апоптоза. Снижение экспрессии генов Cdnf, Bdnf и его рецептора Ntrkb2, а также дофаминовых рецепторов (Drd1, Drd2) у мышей BTBR обнаружено в постнатальный период преимущественно во фронтальной коре, при этом в гиппокампе у половозрелых особей (р60) зафиксировано падение уровня лишь мРНК Drd2. Полученные результаты позволяют предположить, что снижение в постнатальном периоде экспрессии генов Cdnf, Bdnf и Ntrkb2, а также дофаминовых рецепторов во фронтальной коре может приводить к существенным изменениям, характерным для РАС, как морфологии нейронов, так и дофаминовой нейротрансмиссии в корковых структурах мозга. Вместе с тем установленный рост экспрессии p75NTR в критический для развития гиппокампа и фронтальной коры 14-й день эмбриогенеза, возможно, является ключевым для формирования раннего аутизма.

## Introduction

First described back in 1943 (Kanner, 1943), autism (now autism spectrum disorder, ASD) is defined as a group of conditions caused by disorders in prenatal and early postnatal neuroontogenesis that persist throughout a person’s life. ASD is characterized by a decline in the ability to initiate and maintain social interactions and communication, as well as a series of restricted and repetitive inflexible behavior patterns. Data from the Centers for Disease Control and Prevention show a steady increase in the number of children diagnosed with ASD: in 2023, 1 in 36 children was diagnosed, while in 2010, the prevalence of ASD was 1 %. However, according to data from both the WHO and the Ministry of Health of the Russian Federation (Letter of the Ministry of Health No. 15-3/10/1-2140 dated 05/08/2013), the prevalence of ASD was about 1 % of the child population

Currently, there is no unified concept of the pathogenesis of ASD, however, most hypotheses associate the development of this condition with early neurodevelopmental disorders leading to disturbances in mental functions (Hashem et al., 2020). In this regard, special attention when studying the ASD mechanisms is paid to early prenatal brain development (Courchesne et al., 2020). Investigations of induced stem cells obtained from people with ASD have supported the prenatal origin of this disorder (Adhya et al., 2021). A high rate of cell proliferation and a decrease in the degree of differentiation and maturation of GABAergic interneurons have been described (Mariani et al., 2015). Abnormal proliferation and excess prenatal neurogenesis in individuals with ASD appear to explain increases in both cortical neuron numbers (Courchesne et al., 2011) and overall brain mass (Sacco et al., 2015). In addition, the peak expression of most putative risk genes for ASD occurs during the prenatal period (Satterstrom et al., 2020) and is found in a number of brain regions, including cortical areas and the hippocampus (Krishnan et al., 2016; Courchesne et al., 2019). One of the morphological features of ASD is a decrease in volume and sometimes complete agenesis of the corpus callosum (Frazier, Hardan, 2009). At the same time, changes in the volume of the corpus callosum are obviously a consequence of prenatal developmental disorders, since in mammals its formation is completed at the last stage of embryogenesis (Richards et al., 2004).

The neurotransmitter dopamine (DA) is involved in the modulation of learning, reward, and emotional control, which are known to be impaired in autism (Hashem et al., 2020). In people with ASD, a number of polymorphisms are observed in the genes encoding the DA-transporter (DAT) (Dicarlo et al., 2019), DA-metabolic enzymes (Yoo et al., 2013) and DAreceptors (Hettinger et al., 2008; Staal et al., 2015). Moreover, DA neurons derived from pluripotent stem cells from patients with autism are characterized by morphological changes and abnormalities in Ca2+-signal transduction (Nguyen et al., 2018). Based on these data, a connection between the pathogenesis of ASD and dysfunction of the brain DA system has been suggested (Pavăl, 2017).

Neurotrophic factors attract special attention because they play a key role in the regulation of neuronal growth and development, as well as in the control of neuroplasticity (Popova, Naumenko, 2019). Brain-derived neurotrophic factor (BDNF) is one of the most studied neurotrophins that controls synaptogenesis, triggers long-term potentiation, and is involved in memory formation (Castrén, Antila, 2017). An association has been shown between a decreased BDNF blood level in newborns and an increased risk of ASD development (Liu et al., 2021). At the same time, post-mortem studies of the brain of children with ASD have established an increase in the number of prefrontal neurons, which may be a consequence of impaired activity of the BDNF-signal transduction and lead to an excess of axonal connections (Anghelescu, Dettling, 2012). Cerebral dopamine neurotrophic factor (CDNF), first described in 2007, is a non-conventional growth factor and is predominantly localized in the striatum, substantia nigra, hippocampus, cortex and cerebellum (Lindholm et al., 2007, 2008). CDNF is currently being tested in clinical trials as a treatment for Parkinson’s disease (PD) (Lindholm, Saarma, 2022), as it is able to slow down the degeneration of DA neurons (Voutilainen et al., 2011). However, the relationship between neurotrophins and the DA system at different stages of ontogenesis in the context of the development of autism has not been investigated yet.

Based on the stated above, the aim of the current study was to identify the role of the DA system and neurotrophic factors in the development of autism by analyzing the expression patterns of dopamine receptors (Drd1, Drd2) and Cdnf, as well as Bdnf, its receptors (Ntrkb2, Ngfr), and transcription factor Creb1 mediating BDNF effects in the brain structures of BTBR mice, which are known to be a model of autism, in comparison with neurotypical C57Bl/6 J mice at different periods of ontogenesis

## Materials and methods

Experimental animals. The BTBR inbred strain is a widely accepted idiopathic model of autism (Crawley, 2023) as it is characterized by social deficits as well as repetitive behavior (Bolivar et al., 2007; McFarlane et al., 2008). Experiments were conducted on male mice of pathogen-free (SPF) inbred strains BTBR T+tf/J (BTBR) and C57Bl/6 J at the Center for Genetic Resources of Laboratory Animals; Institute of Cytology and Genetics, supported by the Ministry of Science and Higher Education of the Russian Federation (unique identification number: RFMEFI62119X0023). The mice were housed under standard laboratory conditions with a 14-h light cycle, constant humidity (60 %), temperature (23 °C) and with access to balanced food and water ad libitum. All procedures performed with the involvement of laboratory animals were approved by the ethical standards of the Committee on Biological Ethics at the Institute of Cytology and Genetics SB RAS and complied with the ethical standards approved by the legal acts of the Russian Federation (Order of the Ministry of Health of the Russian Federation No. 267 of June 19, 2003), as well as protocols on the treatment of laboratory animals

Experimental design. In autism, the memory consolidation regulated by the hippocampus is impaired, as well as the disturbances observed in executive function and social behavior, for the implementation of which the frontal cortex is mainly responsible. In addition, post-mortem studies of people with ASD revealed a reduced cell size and increased cell density in both the hippocampus and the frontal cortex (Kemper, Bauman, 1998; Courchesne et al., 2011). Based on this, the study of these brain structures in the context of the mechanisms of ASD development was of particular interest.

One of the most variable structures in ASD pathogenesis is the hippocampus, the formation of which begins on the 14th day of embryogenesis (Mangale et al., 2008), while on the 18th day of prenatal development it is already formed (Loones et al., 2000). In addition, on the 17th day of embryogenesis, the corpus callosum is completely formed (Richards et al., 2004); however, the agenesis of the corpus callosum is demonstrated both in BTBR mice (Bohlen et al., 2012) and, often, in people with ASD (Frazier, Hardan, 2009). One of the ASD criteria is hyper- or hyporeactivity to sensory input due to increased or decreased sensitivity to stimuli (DSM-5, ICD-11). Since rodents’ eyes open at 12–13 days and their sensory perception becomes full (Rochefort et al., 2009), it was interesting to study a group of mice at 14 days of age. The juvenile period is an important postnatal stage of development in the study of autism-like behavior, as BTBR mice exhibit low levels of social interaction as early as 28 days after birth (McFarlane et al., 2008). Thus, the following periods of ontogenesis were selected: 14th or 18th day of embryogenesis, as well as 14th, 28th and 60th (reaching maturity) day of postnatal development

Male mice (p14, p28, p60), as well as embryos of the BTBR and C57Bl/6 J strains on the 14th or 18th day of prenatal development, were removed from the experiment by decapitation, and their hippocampi and prefrontal cortices were removed on ice, frozen in liquid nitrogen and stored at –80 °C. For groups e14 and e18, a partial tail biopsy was also performed for subsequent genotyping of the Y chromosome (Sry). The sex of the p14 mice and older was determined by primary sexual characteristics at autopsy. The number of individuals in the experimental group of a certain ontogenetic day (e14, e18, p14, p28, p60) was 10 for each strain.

Obtaining embryos and determining their sex. To obtain embryos in vivo, sexually mature female mice of the BTBR and C57Bl/6 J strains, in a state of estrus, which was determined by analyzing vaginal smears, were mated overnight with males of the corresponding strains. The day of detection of sperm in the vaginal smear was considered the first day of pregnancy. For genotyping of the Y chromosome (Sry), genomic DNA was isolated from embryonic tail tissue by placing it in a lysis solution containing protease K for two hours at 50 °C, followed by extraction in saturated saline according to a previously described protocol (Aljanabi, Martinez, 1997). DNA samples were amplified with primers (see the Table) (Wambach et al., 2014), and PCR products were separated by electrophoresis on a 2 % agarose gel and visualized by ethidium bromide staining. Embryos with the presence of the Y chromosome were used in this work.

**Table 1.**
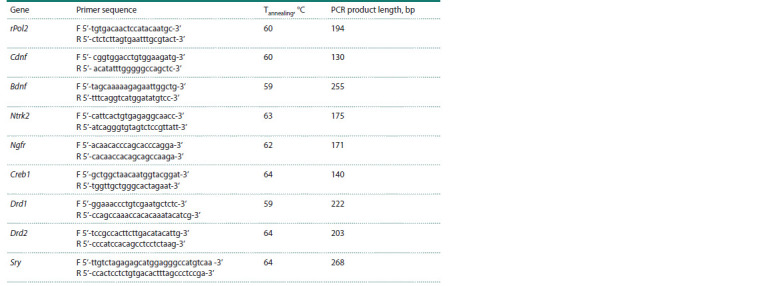
Sequences of the gene-specific oligonucleotide primers and their characteristics

RT-qPCR. Total RNA isolation. Total RNA was isolated with TRIzol Reagent (Life Technologies, USA) as recommended by the manufacturer. Isolated RNA was diluted with water to the concentration of 0.125 μg/μl and stored at –70 °C. The presence of genomic DNA in the RNA preparations was determined as described in (Kulikov et al., 2005; Naumenko, Kulikov, 2006; Naumenko et al., 2008).

Reverse transcription and qPCR. Reverse transcription and real-time PCR were carried out according to the protocol previously described in detail (Kulikov et al., 2005; Naumenko, Kulikov, 2006; Naumenko et al., 2008). Two types of standards were used: external and internal. An internal standard (Polr2a mRNA) was used to monitor reverse transcription and as a basis for calculating the mRNA levels of the studied genes. Mouse DNA with a known concentration (external standard) was used as a PCR control and to determine the copy number of the assayed genes and Polr2a in the samples. Primers used for PCR amplification (see the Table) were developed based on the gene sequences deposited in the Ensembl database and were synthesized by BIOSSET (Russia).

Statistical analysis. The obtained results are presented as mean ± standard error of mean (m ± SEM). For pairwise comparison of means between mouse strains at a certain developmental day, Student’s t-test for independent samples was used. The differences were considered significant at p < 0.05. Normal distribution of the data was verified by the Kolmogorov–Smirnov and Shapiro–Wilk tests. The outliers were identified and excluded by Dixon’s Q test.

## Results

**Fig. 1.**
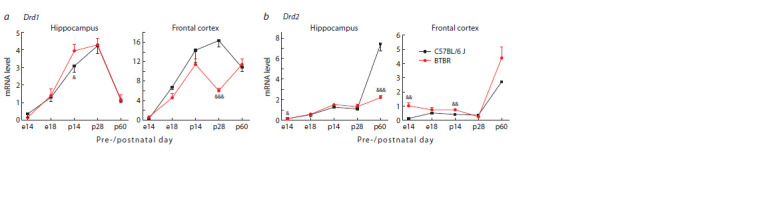
Drd1 (a) and Drd2 (b) mRNA levels in the hippocampus and frontal cortex of BTBR and C57Bl/6 J mice at different periods of pre- and postnatal development Gene expression is presented as the number of cDNA copies per 100 copies of Polr2a cDNA. n = 8–10. Significant difference: & p < 0.05; && p < 0.01; && p < 0.001 – interstrain comparison within a certain day of ontogenesis.

In BTBR mice, a significant increase in the Drd1 mRNA level was revealed in the hippocampus only on the 14th day of postnatal development compared to the C57Bl/6 J strain. A decrease in the expression of this gene was detected in the frontal cortex of BTBR mice on the 28th day of the postnatal period (Fig. 1a). For sexually mature individuals (p60), no interstrain differences in the level of Drd1 expression were observed either in the hippocampus or in the frontal cortex. At the same time, Drd1 gene expression in p60 in the hippocampus of both mouse strains decreased to embryonic values (e18) compared to other stages of postnatal development (p14, p28).

An increase in the Drd2 mRNA level revealed in the frontal cortex of BTBR embryos on the 14th day of prenatal development was leveled out on the 18th day of embryogenesis (Fig. 1b). At the same time, in BTBR mice, an increase in the Drd2 expression was observed in the frontal cortex on the p14 day, while no significant interstrain differences were found upon reaching sexual maturity (p60). Meanwhile, in the hippocampus, interstrain differences in the Drd2 mRNA level were established only in mature mice (p60): in the BTBR strain, a decrease in Drd2 expression was revealed in comparison with the C57Bl/6 J mice, which showed a dramatic increase in the Drd2 mRNA level after the 28th day of the postnatal development.

**Fig. 2.**
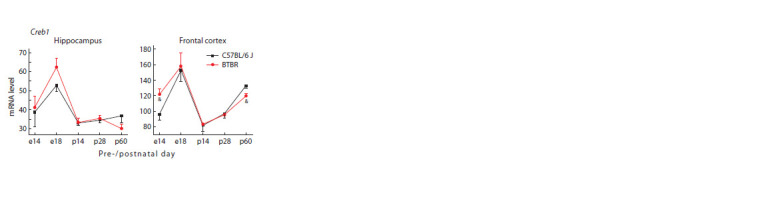
mRNA level of Creb1, encoding the transcription factor CRE-binding protein, in the hippocampus and frontal cortex of BTBR and C57Bl/6 J mice at different periods of pre- and postnatal development. Gene expression is presented as the number of cDNA copies per 100 copies of Polr2a cDNA. n = 8–10. Significant difference: & p < 0.05 – interstrain comparison within a certain day of ontogenesis.

**Fig. 3.**
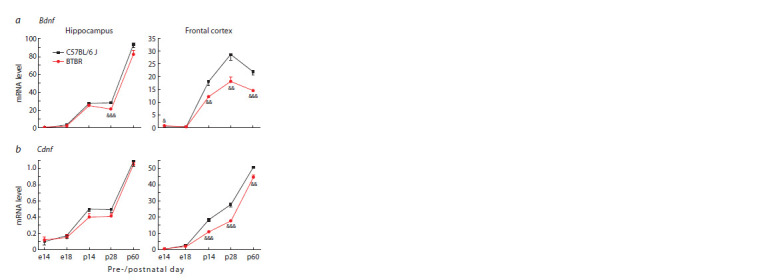
Bdnf (a) and Cdnf (b) mRNA levels in the hippocampus and frontal cortex of BTBR and C57Bl/6 J mice at different periods of pre- and postnatal development Gene expression is presented as the number of cDNA copies per 100 copies of Polr2a cDNA. n = 8–10. Significant difference: && p < 0.01; &&& p < 0.001 – interstrain comparison within a certain day of ontogenesis

In the frontal cortex of the BTBR mice, an increase in the Creb1 mRNA level was revealed on the 14th day of embryogenesis; in contrast, at the age of 60 days in BTBR mice, a decrease in its expression was found (Fig. 2). This dynamics in the Creb1 expression in the BTBR strain is consistent with changes in the cortical Bdnf mRNA level: on the 14th day of embryogenesis, an increase in Bdnf mRNA level was detected, while on the 60th day of postnatal development, a decrease in its expression was shown (Fig. 3a). At the same time, no interstrain differences in the Creb1 expression were detected in the hippocampus during the investigated periods of ontogenesis. Along with this, in the hippocampus and frontal cortex, the peak of Creb1 expression occurred on the 18th day of embryogenesis, while in the frontal cortex, the minimal value of the Creb1 mRNA content was observed on the 14th day of postnatal development (Fig. 2).

**Fig. 4.**
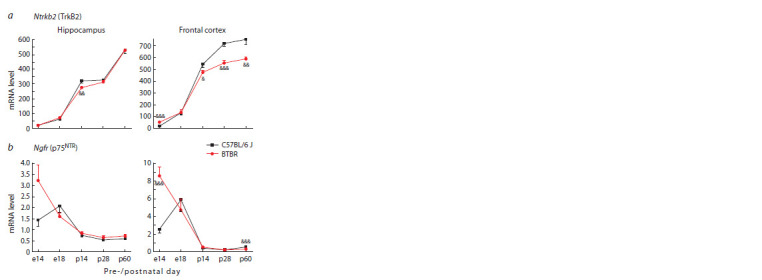
Ntrkb2 (a) and Ngfr (b) mRNA levels in the hippocampus and frontal cortex of BTBR and C57Bl/6 J mice at different periods of pre- and postnatal development. Gene expression is presented as the number of cDNA copies per 100 copies of Polr2a cDNA. n = 8–10. Significant difference: & p < 0.05; && p <0.01; &&& p < 0.001 – interstrain comparison within a certain day of ontogenesis

In BTBR mice, in the frontal cortex during the studied periods of postnatal development (p14, p28, p60), a decrease in the Bdnf coding exon mRNA level was detected, while in the hippocampus, interstrain difference in its expression was revealed only on the 28th day of postnatal development (Fig. 3a). The detected changes in the Bdnf expression in the frontal cortex during the postnatal development of BTBR mice are consistent with the decrease in the Ntrkb2 mRNA level (Fig. 4a), encoding the main BDNF receptor – tyrosine kinase receptor B (TrkB).

Similarly to the changes in the Bdnf mRNA level in the frontal cortex, a decrease in the Cdnf mRNA level on days 14, 28, and 60 of postnatal development was found in BTBR mice (Fig. 3b). At the same time, in the hippocampus, no interstrain differences in the Cdnf expression were observed (Fig. 3b), while, regardless of the mouse strain, a dramatic increase in the Cdnf, Bdnf and Ntrkb2 expression was revealed after day p28.

Analysis of the Ngfr gene (encoding the p75NTR receptor mediating proBDNF action) expression dynamics showed its increase on the 14th day of embryogenesis in BTBR mice both in the hippocampus and frontal cortex (Fig. 4b). At the same time, in the frontal cortex on the 60th day of postnatal development, upon reaching puberty, the Ngfr mRNA level significantly decreased in BTBR mice. In BTBR mice, the peak expression of the Ngfr gene in the investigated brain structures was observed on the 14th day of prenatal development, while in C57Bl/6 J mice, it was revealed on the 18th day of embryogenesis (Fig. 4b), which is in a good agreement with the increase of Creb1 expression (Fig. 2).

## Discussion

People suffering from ASD show changes in neuronal morphology (Minshew, Williams, 2007). In this regard, the analysis of neurotrophins is a promising task for studying the mechanisms of autism. It is known that the BDNF protein is synthesized as a precursor (proBDNF), which is then cleaved to the mature form (mBDNF) (Lessmann et al., 2003). mBDNF is responsible for increasing synaptic plasticity, whereas proBDNF, on the contrary, mediates its decrease (Koshimizu et al., 2009). proBDNF, the expression of which is elevated during the prenatal period (Yang et al., 2009), is a key neurotrophic factor regulating the development of the central nervous system through its influence on neurogenesis (Koshimizu et al., 2009). In addition, increased proBDNF levels were found in post-mortem sections of the fusiform gyrus of people with autism (Garcia et al., 2012), which may be the cause of a decrease in both neuronal differentiation and dendritic spines formation (Teng et al., 2005). BDNF action is mediated by two receptors, namely tyrosine kinase B receptor (TrkB) and p75NTR receptor. p75NTR receptor was previously suggested as a biomarker of ASD, since its mRNA level in peripheral blood was found to be increased in people with autism (Segura et al., 2015)

Here we found that in BTBR mice on the 14th day of embryogenesis in the hippocampus and frontal cortex, when neurogenesis should reach its maximum (Finlay, Darlington, 1995; Chen et al., 2017), the Ngfr gene (encoding p75NTR receptor) mRNA level is increased, while by the 18th day of prenatal development, these differences are already eliminated. Although the p75NTR receptor signal transduction pathways are extremely diverse (Lu et al., 2005), proBDNF binding to p75NTR is known to stimulate cell death (Teng et al., 2005). It can be suggested that in BTBR mice, on the 14th day of prenatal development, apoptotic activity in the hippocampus and frontal cortex is increased, which, together with the impaired synaptogenesis and a decrease in the ability of neurons to form functional connections, leads to behavioral deficits observed in autism (Wei et al., 2014). On the other hand, increased apoptosis can lead to agenesis of the corpus callosum, which is observed in BTBR mice (Bohlen et al., 2012), and often in humans with autism (Frazier, Hardan, 2009). At the same time, in the neurotypical C57Bl/6 J strain, the highest Ngfr mRNA level in the hippocampus and frontal cortex is observed on the 18th day of embryogenesis, which then dramatically decreases together with the decline of neurogenesis in these cell populations (Finlay, Darlington, 1995; Chen et al., 2017). An increase in the p75NTR receptor expression detected in the frontal cortex of BTBR mice on e14 is accompanied by an increase in the mRNA levels of Bdnf coding exon, transcription factor Creb1, and also the Ntrkb2 gene encoding the mBDNF receptor TrkB. Apparently, increased expression of the key elements of the BDNF-TrkB signaling pathway is a compensatory response to the putative increase of apoptotic activity mediated by p75NTR receptor, since BDNF, when bound to TrkB, triggers protein synthesis, growth and maturation of neurons (Fenner, 2012). At the same time, in the frontal cortex of BTBR mice, a decrease in both Bdnf and its receptor Ntrkb2 (TrkB) mRNA levels was observed from day p14 to sexual maturity. That may indicate a decrease in neuroprotective and neurotrophic properties in cortical structures of BTBR mice and lead to autism-like behavior. This, in accordance with our previous data, showed that BDNF overexpression in the hippocampus reduces anxiety and stereotypic behavior in BTBR mice, which are among the diagnostic criteria for ASD (Ilchibaeva et al., 2023).

Earlier it was found that Cc2d1a/Freud-1 knockdown in the hippocampus of BTBR mice did not affect spatial memory and phosphorylation of the CREB transcription factor (Belokopytova et al., 2022), although such an effect was found in C57Bl/6 J mice (Kondaurova et al., 2021). Based on these data, we suggested that the functional activity of the CREB transcription factor (stimulating BDNF expression) is impaired in BTBR mice, which may be the cause of the disturbances in the BDNF signaling cascade identified in the current study.

ASD is often accompanied by dysfunction in certain neurotransmitter systems (Rodnyy et al., 2023). In particular, increased levels of serotonin (Pourhamzeh et al., 2022), as well as disturbances in the brain DA system functioning, manifesting in changes in both DA metabolism (Yoo et al., 2013) and signal transduction (Staal et al., 2015; DiCarlo et al., 2019), have been established in ASD. It is known that DA can affect cell proliferation and differentiation of telencephalon cells during embryonic development: blockade of DA-type 1 receptor (D1R) leads to a decrease in the rate of cell division, while stimulation of DA-type 2 receptor (D2R), on the contrary, promotes its activation (Popolo et al., 2004). On the other hand, transduction of the DA signal upon D2R activation reduces the migration of GABAergic interneurons in the telencephalon (Crandall et al., 2007), which can lead to disruption of inhibitory processes in cortical areas observed in BTBR mice (Cellot et al., 2016) and often in people with autism (Enticott et al., 2013). These data are in a good agreement with our results identifying an increase in the Drd2 mRNA level in the frontal cortex and hippocampus of BTBR mice on the 14th day of prenatal development, which may likely contribute to disturbances in the formation and functioning of these brain structures

There is a large amount of data on the relationship between the BDNF and DA systems. For example, BDNF has been proposed as a promising agent in the treatment of Parkinson’s disease, given its stimulatory effect on both DA release (Neal et al., 2003) and the overall trophic effect on DA neurons (Palasz et al., 2020) upon activation of Bdnf expression (Küppers, Beyer, 2001). In this regard, unidirectional changes in the expression of both D2R and BDNF-TrkB detected on the 14th day of embryogenesis in BTBR mice are consistent with the concept of the relationship between the DA and BDNF systems. At the same time, the decrease in the Drd2 mRNA level observed in the hippocampus of BTBR mice at the age of 60 days together with the absence of changes in the expression of genes encoding the studied neurotrophic factors may be a consequence of a disruption in the CREB-dependent signaling pathway (Belokopytova et al., 2022) and lead to deficits in memory and learning. At the same time, the hippocampal and cortical Drd1 expression did not change in the BTBR strain during the studied periods of embryogenesis. However, in the postnatal period, interstrain differences in the Drd1 mRNA levels were detected both in the hippocampus and frontal cortex. The Drd1 expression level in the hippocampus of BTBR mice was increased on day p14, while in the frontal cortex, on the contrary, its decrease was found already in the juvenile period (p28). Despite the absence of interstrain differences in the Drd1 expression level upon reaching sexual maturity (p60), the observed changes in the Drd1 mRNA levels during critical periods after birth may be among the reasons for learning and memory impairments observed in BTBR mice already in juvenile age (McFarlane et al., 2008). Since the detected decrease in the Drd1 mRNA level in the frontal cortex of BTBR mice on the 28th day after birth was accompanied by a decrease in the Bdnf and Ntrkb2 (TrkB) expression, the participation of the BDNF-TrkB signaling pathway in the regulation of D1R expression was suggested. This is in good agreement with our results showing that Bdnf overexpression in the hippocampus of BTBR mice leads to an increase in the Drd1 gene expression along with a decrease in anxiety and stereotypy (Ilchibaeva et al., 2023).

The regulation of DA neurotransmission is also often associated with the recently discovered non-canonical neurotrophin CDNF that has neuroprotective properties in conditions associated with degeneration of DA neurons (Voutilainen et al., 2011). There are currently no data on the role of CDNF in the pathogenesis of ASD. Here we showed for the first time the decrease in the Cdnf expression in the frontal cortex of BTBR mice throughout the entire studied period of postnatal development, starting with eye opening at day p14 till the onset of sexual maturity at day p60. Considering that CDNF is characterized by anti-apoptotic and neurotrophic effects (Boћok et al., 2018), it was suggested that there is an increased risk of cell death activation as well as reduction of cytoprotective properties in the frontal cortex of BTBR mice during the postnatal development, which may lead, among other things, to disturbances in DA neurotransmission and, hence, manifestation of autism-like behavior

## Conclusion

Thus, in the hippocampus and frontal cortex of BTBR mice, characterized by autism-like behavior, a significant dysregulation of the expression patterns of Cdnf, key DA receptors, Bdnf and its receptors, as well as the transcription factor CREB was shown. It was suggested that the identified disturbances in the expression of the studied genes on the 14th day of embryogenesis are critical for the formation of an autism-like phenotype. The decrease in the expression of Cdnf, as well as Bdnf and its receptor Ntrkb2 in the frontal cortex during the studied periods of postnatal development apparently results in critical changes in the morphology of neurons in cortical brain regions. At the same time, the revealed decrease in Drd2 gene expression in the postnatal period may be associated with learning and memory impairments observed in BTBR mice

## Conflict of interest

The authors declare no conflict of interest.

## References

Adhya D., Swarup V., Nagy R., Dutan L., Shum C., Valencia-Alarcуn
E.P., Jozwik K.M., Mendez M.A., Horder J., Loth E., Nowosiad
P., Lee I., Skuse D., Flinter F.A., Murphy D., McAlonan G.,
Geschwind
D.H., Price J., Carroll J., Srivastava D.P., Baron-Cohen
S. Atypical neurogenesis in induced pluripotent stem cells from
autistic individuals. Biol. Psychiatry. 2021;89(5):486-496. DOI
10.1016/j.biopsych.2020.06.014

Aljanabi S.M., Martinez I. Universal and rapid salt-extraction of high
quality genomic DNA for PCR-based techniques. Nucleic Acids Res.
1997;25(22):4692-4693. DOI 10.1093/nar/25.22.4692

Anghelescu I., Dettling M. Neuron number in children with autism.
JAMA. 2012;307(8):783. DOI 10.1001/jama.2012.191

Belokopytova I.I., Kondaurova E.M., Kulikova E.A., Ilchibaeva T.V.,
Naumenko V.S., Popova N.K. Effects of the Cc2d1a/Freud-1 knockdown
in the hippocampus of BTBR mice on the autistic-like behavior,
expression of serotonin 5-HT1A and D2 dopamine receptors,
and CREB and NF-κB intracellular signaling. Biochemistry (Mosc.).
2022;87(10):1206-1218. DOI 10.1134/S0006297922100145

Bohlen M.O., Bailoo J.D., Jordan R.L., Wahlsten D. Hippocampal commissure
defects in crosses of four inbred mouse strains with absent
corpus callosum. Genes Brain Behav. 2012;11(7):757-766. DOI
10.1111/j.1601-183X.2012.00802.x

Boћok V., Yu L.Y., Palgi J., Arumӓe U. Antioxidative CXXC peptide
motif from mesencephalic astrocyte-derived neurotrophic factor antagonizes
programmed cell death. Front. Cell Dev. Biol. 2018;6:106.
DOI 10.3389/fcell.2018.00106

Bolivar V.J., Walters S.R., Phoenix J.L. Assessing autism-like behavior
in mice: variations in social interactions among inbred strains. Behav.
Brain Res. 2007;176(1):21-26. DOI 10.1016/j.bbr.2006.09.007

Castrén E., Antila H. Neuronal plasticity and neurotrophic factors
in drug responses. Mol. Psychiatry. 2017;22(8):1085-1095. DOI
10.1038/mp.2017.61

Cellot G., Maggi L., Di Castro M.A., Catalano M., Migliore R., Migliore
M., Scattoni M.L., Calamandrei G., Cherubini E. Premature
changes in neuronal excitability account for hippocampal network
impairment and autistic-like behavior in neonatal BTBR T+tf/J
mice. Sci. Rep. 2016;6:31696. DOI 10.1038/srep31696

Chen V.S., Morrison J.P., Southwell M.F., Foley J.F., Bolon B., Elmore
S.A. Histology atlas of the developing prenatal and postnatal
mouse central nervous system, with emphasis on prenatal days
E7.5 to E18.5. Toxicol. Pathol. 2017;45(6):705-744. DOI 10.1177/
0192623317728134

Courchesne E., Mouton P.R., Calhoun M.E., Semendeferi K., Ahrens-
Barbeau C., Hallet M.J., Barnes C.C., Pierce K. Neuron number
and size in prefrontal cortex of children with autism. JAMA. 2011;
306(18):2001-2010. DOI 10.1001/jama.2011.1638

Courchesne E., Pramparo T., Gazestani V.H., Lombardo M.V., Pierce
K., Lewis N.E. The ASD Living Biology: from cell proliferation
to clinical phenotype. Mol. Psychiatry. 2019;24(1):88-107. DOI
10.1038/s41380-018-0056-y

Courchesne E., Gazestani V.H., Lewis N.E. Prenatal origins of ASD:
The when, what, and how of ASD development. Trends Neurosci.
2020;43(5):326-342. DOI 10.1016/j.tins.2020.03.005

Crandall J.E., McCarthy D.M., Araki K.Y., Sims J.R., Ren J.Q.,
Bhide P.G. Dopamine receptor activation modulates GABA neuron
migration from the basal forebrain to the cerebral cortex. J. Neurosci.
2007;27(14):3813-3822. DOI 10.1523/JNEUROSCI.5124-
06.2007

Crawley J.N. Twenty years of discoveries emerging from mouse models
of autism. Neurosci. Biobehav. Rev. 2023;146:105053. DOI
10.1016/j.neubiorev.2023.105053

DiCarlo G.E., Aguilar J.I., Matthies H.J., Harrison F.E., Bundschuh
K.E., West A., Hashemi P., Herborg F., Rickhag M., Chen H.,
Gether U., Wallace M.T., Galli A. Autism-linked dopamine transporter
mutation alters striatal dopamine neurotransmission and dopamine-
dependent behaviors. J. Clin. Invest. 2019;129(8):3407-
3419. DOI 10.1172/JCI127411

Enticott P.G., Kennedy H.A., Rinehart N.J., Tonge B.J., Bradshaw J.L.,
Fitzgerald P.B. GABAergic activity in autism spectrum disorders:
an investigation of cortical inhibition via transcranial magnetic
stimulation. Neuropharmacology. 2013;68:202-209. DOI 10.1016/
j.neuropharm.2012.06.017

Fenner B.M. Truncated TrkB: beyond a dominant negative receptor.
Cytokine Growth Factor Rev. 2012;23(1-2):15-24. DOI 10.1016/
j.cytogfr.2012.01.002

Finlay B.L., Darlington R.B. Linked regularities in the development
and evolution of mammalian brains. Science. 1995;268(5217):
1578-1584. DOI 10.1126/science.7777856

Frazier T.W., Hardan A.Y. A meta-analysis of the corpus callosum
in autism. Biol. Psychiatry. 2009;66(10):935-941. DOI 10.1016/
j.biopsych.2009.07.022

Garcia K.L., Yu.G., Nicolini C., Michalski B., Garzon D.J., Chiu V.S.,
Tongiorgi E., Szatmari P., Fahnestock M. Altered balance of proteolytic
isoforms of pro-brain-derived neurotrophic factor in autism.
J. Neuropathol. Exp. Neurol. 2012;71(4):289-297. DOI 10.1097/
NEN.0b013e31824b27e4

Hashem S., Nisar S., Bhat A.A., Yadav S.K., Azeem M.W., Bagga P.,
Fakhro K., Reddy R., Frenneaux M.P., Haris M. Genetics of structural
and functional brain changes in autism spectrum disorder. Transl.
Psychiatry. 2020;10(1):229. DOI 10.1038/s41398-020-00921-3

Hettinger J.A., Liu X., Schwartz C.E., Michaelis R.C., Holden J.J.
A DRD1 haplotype is associated with risk for autism spectrum disorders
in male-only affected sib-pair families. Am. J. Med. Genet. B
Neuropsychiatr. Genet. 2008;147B(5):628-636. DOI 10.1002/ajmg.b.
30655

Ilchibaeva T., Tsybko A., Lipnitskaya M., Eremin D., Milutinovich K.,
Naumenko V., Popova N. Brain-derived neurotrophic factor (BDNF)
in mechanisms of autistic-like behavior in BTBR mice: crosstalk
with the dopaminergic brain system. Biomedicines. 2023;11(5):1482.
DOI 10.3390/biomedicines11051482

Kanner L. Autistic disturbances of affective contact. Nervous Child.
1943;2:217-250

Kemper T.L., Bauman M. Neuropathology of infantile autism. J. Neuropathol.
Exp. Neurol. 1998;57(7):645-652. DOI 10.1097/00005072-
199807000-00001

Kondaurova E.M., Plyusnina A.V., Ilchibaeva T.V., Eremin D.V., Rodnyy
A.Y., Grygoreva Y.D., Naumenko V.S. Effects of a Cc2d1a/
Freud-1 knockdown in the hippocampus on behavior, the serotonin
system, and BDNF. Int. J. Mol. Sci. 2021;22(24):13319. DOI
10.3390/ijms222413319

Koshimizu H., Kiyosue K., Hara T., Hazama S., Suzuki S., Uegaki K.,
Nagappan G., Zaitsev E., Hirokawa T., Tatsu Y., Ogura A., Lu B.,
Kojima M. Multiple functions of precursor BDNF to CNS neurons:
negative regulation of neurite growth, spine formation and cell survival.
Mol. Brain. 2009;2:27. DOI 10.1186/1756-6606-2-27

Krishnan A., Zhang R., Yao V., Theesfeld C.L., Wong A.K., Tadych A.,
Volfovsky N., Packer A., Lash A., Troyanskaya O.G. Genome-wide
prediction and functional characterization of the genetic basis of
autism
spectrum disorder. Nat. Neurosci. 2016;19(11):1454-1462.
DOI 10.1038/nn.4353

Kulikov A.V., Naumenko V.S., Voronova I.P., Tikhonova M.A., Popova
N.K. Quantitative RT-PCR assay of 5-HT1A and 5-HT2A serotonin
receptor mRNAs using genomic DNA as an external standard.
J. Neurosci. Methods. 2005;141(1):97-101. DOI 10.1016/
j.jneumeth.2004.06.005

Küppers E., Beyer C. Dopamine regulates brain-derived neurotrophic
factor (BDNF) expression in cultured embryonic mouse striatal
cells. Neuroreport. 2001;12(6):1175-1179. DOI 10.1097/00001756-
200105080-00025

Lessmann V., Gottmann K., Malcangio M. Neurotrophin secretion: current
facts and future prospects. Prog. Neurobiol. 2003;69(5):341-
374. DOI 10.1016/s0301-0082(03)00019-4

Lindholm P., Saarma M. Cerebral dopamine neurotrophic factor protects
and repairs dopamine neurons by novel mechanism. Mol. Psychiatry.
2022;27(3):1310-1321. DOI 10.1038/s41380-021-01394-6

Lindholm P., Voutilainen M.H., Laurén J., Peränen J., Leppänen V.M.,
Andressoo J.O., Lindahl M., Janhunen S., Kalkkinen N., Timmusk
T., Tuominen R.K., Saarma M. Novel neurotrophic factor
CDNF protects and rescues midbrain dopamine neurons in vivo.
Nature.
2007;448(7149):73-77. DOI 10.1038/nature05957

Lindholm P., Peränen J., Andressoo J.O., Kalkkinen N., Kokaia Z.,
Lindvall O., Timmusk T., Saarma M. MANF is widely expressed
in mammalian tissues and differently regulated after ischemic and
epileptic insults in rodent brain. Mol. Cell. Neurosci. 2008;39(3):
356-371. DOI 10.1016/j.mcn.2008.07.016

Liu S.H., Shi X.J., Fan F.C., Cheng Y. Peripheral blood neurotrophic
factor levels in children with autism spectrum disorder: a meta-analysis.
Sci. Rep. 2021;11(1):15. DOI 10.1038/s41598-020-79080-w

Loones M.T., Chang Y., Morange M. The distribution of heat shock
proteins in the nervous system of the unstressed mouse embryo
suggests a role in neuronal and non-neuronal differentiation. Cell
Stress Chaperones. 2000;5(4):291-305. DOI 10.1379/1466-1268
(2000)005<0291:tdohsp>2.0.co;2

Lu B., Pang P.T., Woo N.H. The yin and yang of neurotrophin action.
Nat. Rev. Neurosci. 2005;6(8):603-614. DOI 10.1038/nrn1726

Mangale V.S., Hirokawa K.E., Satyaki P.R., Gokulchandran N.,
Chikbire S., Subramanian L., Shetty A.S., Martynoga B., Paul J.,
Mai M.V., Li Y., Flanagan L.A., Tole S., Monuki E.S. Lhx2 selector
activity specifies cortical identity and suppresses hippocampal organizer
fate. Science. 2008;319(5861):304-309. DOI 10.1126/science.
1151695

Mariani J., Coppola G., Zhang P., Abyzov A., Provini L., Tomasini L.,
Amenduni M., Szekely A., Palejev D., Wilson M., Gerstein M., Grigo-
renko
E.L., Chawarska K., Pelphrey K.A., Howe J.R., Vaccarino F.M.
FOXG1-dependent dysregulation of GABA/glutamate neuron differentiation
in autism spectrum disorders. Cell. 2015;162(2):375-
390. DOI 10.1016/j.cell.2015.06.034

McFarlane H.G., Kusek G.K., Yang M., Phoenix J.L., Bolivar V.J.,
Crawley J.N. Autism-like behavioral phenotypes in BTBR T+tf/J
mice. Genes Brain Behav. 2008;7(2):152-163. DOI 10.1111/j.1601-
183X.2007.00330.x

Minshew N.J., Williams D.L. The new neurobiology of autism: cortex,
connectivity, and neuronal organization. Arch. Neurol. 2007;64(7):
945-950. DOI 10.1001/archneur.64.7.945

Naumenko V.S., Kulikov A.V. Quantitative assay of 5-HT1A receptor
gene expression in the brain. Mol. Biol. (Mosk.). 2006;40(1):30-36.
DOI 10.1134/S0026893306010067

Naumenko V.S., Osipova D.V., Kostina E.V., Kulikov A.V. Utilization
of a two-standard system in real-time PCR for quantification of gene
expression in the brain. J. Neurosci. Methods. 2008;170(2):197-203.
DOI 10.1016/j.jneumeth.2008.01.008

Neal M., Cunningham J., Lever I., Pezet S., Malcangio M. Mechanism
by which brain-derived neurotrophic factor increases dopamine
release from the rabbit retina. Invest. Ophthalmol. Vis. Sci. 2003;
44(2):791-798. DOI 10.1167/iovs.02-0557

Nguyen H.T.N., Kato H., Masuda K., Yamaza H., Hirofuji Y., Sato H.,
Pham T.T.M., Takayama F., Sakai Y., Ohga S., Taguchi T., Nonaka
K. Impaired neurite development associated with mitochondrial
dysfunction in dopaminergic neurons differentiated from exfoliated
deciduous tooth-derived pulp stem cells of children with autism
spectrum disorder. Biochem. Biophys. Rep. 2018;16:24-31. DOI
10.1016/j.bbrep.2018.09.004

Palasz E., Wysocka A., Gasiorowska A., Chalimoniuk M., Niewiadomski
W., Niewiadomska G. BDNF as a promising therapeutic agent in
Parkinson’s disease. Int. J. Mol. Sci. 2020;21(3):1170. DOI 10.3390/
ijms21031170

Pavăl D. A Dopamine hypothesis of autism spectrum disorder. Dev.
Neurosci. 2017;39(5):355-360. DOI 10.1159/000478725

Popolo M., McCarthy D.M., Bhide P.G. Influence of dopamine on precursor
cell proliferation and differentiation in the embryonic mouse
telencephalon. Dev. Neurosci. 2004;26(2-4):229-244. DOI 10.1159/
00008214

Popova N.K., Naumenko V.S. Neuronal and behavioral plasticity:
the role of serotonin and BDNF systems tandem. Expert Opin.
Ther. Targets. 2019;23(3):227-239. DOI 10.1080/14728222.2019.
1572747

Pourhamzeh M., Moravej F.G., Arabi M., Shahriari E., Mehrabi S.,
Ward R., Ahadi R., Joghataei M.T. The roles of serotonin in neuropsychiatric disorders. Cell. Mol. Neurobiol. 2022;42(6):1671-1692.
DOI 10.1007/s10571-021-01064-9

Richards L.J., Plachez C., Ren T. Mechanisms regulating the development
of the corpus callosum and its agenesis in mouse and human.
Clin. Genet. 2004;66(4):276-289. DOI 10.1111/j.1399-0004.2004.
00354.x

Rochefort N.L., Garaschuk O., Milos R.I., Narushima M., Marandi N.,
Pichler B., Kovalchuk Y., Konnerth A. Sparsification of neuronal activity
in the visual cortex at eye-opening. Proc. Natl. Acad. Sci. USA.
2009;106(35):15049-15054. DOI 10.1073/pnas.0907660106

Rodnyy A.Y., Kondaurova E.M., Tsybko A.S., Popova N.K., Kudlay
D.A., Naumenko V.S. The brain serotonin system in autism. Rev.
Neurosci. 2023;35(1):1-20. DOI 10.1515/revneuro-2023-0055

Sacco R., Gabriele S., Persico A.M. Head circumference and brain
size in autism spectrum disorder: A systematic review and metaanalysis.
Psychiatry Res. 2015;234(2):239-251. DOI 10.1016/
j.pscychresns.2015.08.016

Satterstrom F.K., Kosmicki J.A., Wang J., Breen M.S., De Rubeis S.,
An J.Y., Peng M., … Guerrero E.E., Dias C.; Autism Sequencing
Consortium; iPSYCH-Broad Consortium; Betancur C., Cook E.H.,
Gallagher L., Gill M., Sutcliffe J.S., Thurm A., Zwick M.E., Børglum
A.D., State M.W., Cicek A.E., Talkowski M.E., Cutler D.J.,
Devlin B., Sanders S.J., Roeder K., Daly M.J., Buxbaum J.D.
Large-scale exome sequencing study implicates both developmental
and functional changes in the neurobiology of autism. Cell. 2020;
180(3):568-584.e23. DOI 10.1016/j.cell.2019.12.036

Segura M., Pedreño C., Obiols J., Taurines R., Pаmias M., Grünblatt E.,
Gella A. Neurotrophin blood-based gene expression and social cognition
analysis in patients with autism spectrum disorder.
Neurogenetics.
2015;16(2):123-131. DOI 10.1007/s10048-014-0434-9

Staal W.G., Langen M., van Dijk S., Mensen V.T., Durston S. DRD3
gene and striatum in autism spectrum disorder. Br. J. Psychiatry.
2015;206(5):431-432. DOI 10.1192/bjp.bp.114.148973

Teng H.K., Teng K.K., Lee R., Wright S., Tevar S., Almeida R.D., Kermani
P., Torkin R., Chen Z.Y., Lee F.S., Kraemer R.T., Nykjaer A.,
Hempstead B.L. ProBDNF induces neuronal apoptosis via activation
of a receptor complex of p75NTR and sortilin. J. Neurosci.
2005;25(22):5455-5463. DOI 10.1523/JNEUROSCI.5123-04.2005

Voutilainen M.H., Bäck S., Peränen J., Lindholm P., Raasmaja A., Männistö
P.T., Saarma M., Tuominen R.K. Chronic infusion of CDNF
prevents 6-OHDA-induced deficits in a rat model of Parkinson’s
disease. Exp. Neurol. 2011;228(1):99-108. DOI 10.1016/j.exp
neurol.2010.12.013

Wambach C.M., Patel S.N., Kahn D.A. Maternal and fetal factors that
contribute to the localization of T regulatory cells during pregnancy.
Am. J. Reprod. Immunol. 2014;71(5):391-400. DOI 10.1111/aji.
12223

Wei H., Alberts I., Li X. The apoptotic perspective of autism. Int. J.
Dev. Neurosci. 2014;36:13-18. DOI 10.1016/j.ijdevneu.2014.04.004

Yang J., Siao C.J., Nagappan G., Marinic T., Jing D., McGrath K.,
Chen Z.Y., Mark W., Tessarollo L., Lee F.S., Lu B., Hempstead B.L.
Neuronal release of proBDNF. Nat. Neurosci. 2009;12(2):113-115.
DOI 10.1038/nn.2244

Yoo H.J., Cho I.H., Park M., Yang S.Y., Kim S.A. Association of the
catechol-o-methyltransferase gene polymorphisms with Korean
autism
spectrum disorders. J. Korean Med. Sci. 2013;28(9):1403-
1406. DOI 10.3346/jkms.2013.28.9.1403

